# Dietary High Sodium Fluoride Impairs Digestion and Absorption Ability, Mucosal Immunity, and Alters Cecum Microbial Community of Laying Hens

**DOI:** 10.3390/ani10020179

**Published:** 2020-01-21

**Authors:** Liping Miao, Mingkun Zhu, Huaiyu Li, Qianqian Xu, Xinyang Dong, Xiaoting Zou

**Affiliations:** Key Laboratory of Animal Feed and Nutrition of Zhejiang Province, Key Laboratory of Animal Nutrition and Feed Science in East China, Ministry of Agriculture, The Key Laboratory of Molecular Animal Nutrition, Ministry of Education, College of Animal Sciences, Zhejiang University, Hangzhou 310058, China; Lipmiao@163.com (L.M.); mkzhu@zju.edu.cn (M.Z.); 21817082@zju.edu.cn (H.L.); qianqianxu@zju.edu.cn (Q.X.); sophiedxy@zju.edu.cn (X.D.)

**Keywords:** fluoride, laying hen, digestion and absorption ability, mucosal immunity, cecum microbe

## Abstract

**Simple Summary:**

In the current study, the effects of dietary fluoride (F) on tissue retention, digestive enzymes activities, mucosal immunity, and cecum microbial community of laying hens were investigated. Results revealed that dietary high F intake increased the concentrations of F in duodenum, decreased intestinal digestive enzymes activities, reduced intestine mucosal immunity, and disturbed the composition of cecum microbiota.

**Abstract:**

(1) Background: This study was conducted to investigate the effects of dietary fluoride (F) on tissue retention, digestive enzymes activities, mucosal immunity, and cecum microbial community of laying hens. (2) Methods: Total of 288 37-week-old Hy-Line Gray laying hens with similar laying rate (85.16% ± 3.87%) were adapted to the basal diets for ten days, and then allocated into three groups at random (*n* = 9, 6, 6 replicates/group). The concentrations of F in the diets were 31.19 (the control group, CON), 431.38 (F400, low-F group) and 1237.16 mg/kg (F1200, high-F group), respectively. The trial lasted for 59 days. (3) Results: Results suggested that F residuals in duodenum responded to dietary F concentrations positively. The activities of amylase, maltase and lactase were decreased in high-F group, compared with those in the control group. The mRNA expression levels of jejunum and ileum secretory immunoglobulin A (sIgA) and Mucin 2, and sIgA concentrations were decreased inhigh-F group, than those in the control group. The observed operational taxonomic units (OTUs) of laying hens in high-F group were higher than the CON and low-F groups, and the bacterial structure was different from the other two groups. The *Lactobacillus* was higher in the control group, while *Gammaproteobacteria*, *Escherichia-Shigella*, *Streptococcaceae*, and *Enterobacteriaceae* were higher in the high-F group. (4) Conclusions: The actual results confirmed that dietary high F intake increased the F residuals in duodenum, and reduced the digestion and absorption of nutrients and immunity via decreasing the activities of digestive enzymes, impairing intestine mucosal immunity, and disturbing the cecum microbial homeostasis of laying hens.

## 1. Introduction

Fluoride (F), the compound of fluorine (the most active element in the natural environment), exists very extensively in the living environment, for instance, groundwater, soil, food, industrial pollutant, drugs, and dental hygiene products [1,2]. As we all know that F is necessary in lots of physiological activities. However, previous literatures have demonstrated that over-consumption of F not only damaged hard tissues, such as bone [3,4] and tooth [5,6], but also soft tissues, such as liver, kidney, thyroid, testis, uterus, and ovary [7,8,9,10,11]. Recently, literatures have suggested that over-intake F had intestinal toxicity [12,13], while the underlying mechanisms of the intestinal toxicity of F are unclear. Hence, it is necessary to explore the intestinal toxicity of F using an effectual animal model. Laying hens, one of the most resistant to F, were used to investigate the intestinal toxicity of F in the present study. Previous research reported that F was absorbed in the whole gastrointestinal tract (from stomach to colon) proportionally to luminal concentration F (the form of NaF, 0.5–10 mM) via a passive diffusion, whereas a back diffusion placed when serosal concentration passed a certain level [14]. It was hypothesized that high F concentration in the gastrointestinal tract would damage the intestine and other tissues.

The intestines (large and small) play a vital role in digesting and absorbing nutrients in diet, maintaining the homeostasis of electrolyte, and represent the largest compartment of the immune system [15]. It is constantly exposed to a changing intraluminal environment and has the capacity to adapt its structure and function to variations in the diet [16]. For instance, starvation can swiftly decrease the density of villi, and reduce villus length, cause microvilli clustering, and accelerate epithelial cell turnover [17,18]. Hence, it is supposed that constant exposure to dietary high F level may alter intestinal structure and function of laying hens. In another way, the gut microbiota can be taken as an extension of the self, regulates immune and inflammatory response, and diet can alter the composition of the microbiota [19,20]. However, the relationship between the dietary F level and the gut microbiota is unclear. In the current study, fluoride-exposed laying hen model was established via feeding diet with F and used to explore the F residual in duodenum, and the alteration of the digestive enzymes activities, mucosal immunity, and cecal microbiota community.

## 2. Materials and Methods

### 2.1. Animal Ethics

The current study was carried out according to the Chinese guidelines for animal welfare and approved by the Animal Welfare Committee of Zhejiang University (No. ZJU2013105002, Hangzhou, China). The birds used in the current study were treated humanely. The great efforts were made to minimize their suffering.

### 2.2. Animals and Sample Collection

We acclimated 288 healthy Hy-Line Gray hens (37 weeks, Hangzhou, China) to the basal diets for 10 days. Then, birds were arbitrarily allocated to three groups (96 hens per group, six replicates), which included the control group fed a basal diet (control), or a basal diet supplemented with 400, and 1200 mg F/kg feed from sodium fluoride (NaF, analytical reagent grade, Sinopharm Chemical Reagent Co., Ltd., Shanghai, China). The actual contents of dietary F levels were 31.19 (the control group, CON), 431.38 (low-F, F400 treatment) and 1237.16 mg/kg (high-F, F1200 treatment), respectively (with a potentiometric method using an ion selective electrode (Leici, PF-1 01, Shanghai, China)) [21]. The diets were formulated according to the NY/T 33-2004 (Chicken Feeding Standard, Agricultural Industry Standard of the P. R. China.). The ingredients and analysis of basal diets were listed in Table 1. The trial lasted for 59 days (10-day adaptation period and 49-day experimental stage). Hens were housed in three-layer cages of the full scale in a ventilated room (4 hens in an individual cage equipped with two nipple drinkers) with average temperature at 26 ± 2 °C, relative humidity at 65% ± 5%, and a 16:8 h light:dark cycle. The diets and water were free to access for all hens at all times during the trial. All birds were treated according to the criteria outlined in the “Guide for the Care and Use of Laboratory Animals” published by the National Institute of Health.

Twelve hens from each treatment were randomly euthanized with pentobarbital sodium and sacrificed on the 59th days of the trial. The small intestine segments and five luminal cecum contents were collected immediately and stored in liquid nitrogen. The intestinal contents and mucosa were collected from duodenum for digestive enzymes activities determination.

### 2.3. Experimental Parameters Measured

The residuals of F in the duodenum were measured by a potentiometric method using an ion selective electrode (Leici, PF-1 01, Shanghai, China) [21]. Digestive enzymes activities (maltase, sucrase, lactase, lipase, amylase, and trypsin) were measured and calculated following with the protocols of commercial kits (Nanjing Jiancheng Bioengineering Institute, Nanjing, China). Secretory immunoglobulin A (sIgA) concentrations in jejunum and ileum were measured by ELISA kits (Shanghai Mlbio Institute, Shanghai, China).

### 2.4. Real-Time PCR

Total RNA from small intestines was extracted by Trizol reagent according to the manufacturer’s instructions (Invitrogen), and dissolved in 20 μL diethylpyrocarbonate-treated water. Subsequently, the concentrations and purities were measured at 260/280 nm (ratio *>* 1.9) using Nano Drop Spectrophotometer (ND-2000; Gene Company Ltd.). Reverse transcription steps of complementary DNA were carried out according to the manufacturer’s instructions (Vazyme Biotechnology, Nanjing, Jiangsu, China). Primer sequences used in the study were synthesized and presented in Table 2 (Generay Biotech Co., Ltd., Shanghai, China). The real-time quantitative PCR reactions were taken place with a SYBR Premix PCR kit (Vazyme Biotechnology, Nanjing, Jiangsu, China) according to the instructions. 18s rRNA was taken as an endogenous reference gene. The relative abundances of genes were analysised with the 2-^ΔΔCt^ method.

### 2.5. DNA Extraction and Quantitative PCR Amplification

Total microbial genomic DNA was extracted from the cecum contents of five hens from each treatment using the QIAamp DNA Stool Mini Kit (QIAGEN, CA). The concentration and quality of the extracted DNA were assessed with 1% agarose gels electrophoresis and a NanoDrop ND-2000 spectrophotometer (ND-2000; Gene Company Ltd.). The V3-V4 hyper-variable region of the 16S rRNA genes was amplified by specific degenerate primers (341F: 5′-CCTAYGGGRBGCASCAG-3′, 806R: 5′-GGACTACNNGGGTATCTAAT-3′). The PCR program was conducted with ABI GeneAmp^®^ PCR System 9700 (Applied Biosystems Life Technologies, Foster City, CA, USA), following the conditions: 98 °C for 1 min, 30 cycles 98 °C for 10 s, 50 °C for 30 s, 72 °C for 30 s, and last 72 °C for 5 min. The gene library was built with the Illumina TruSeq DNA PCR-Free Library preparation kit, and sequenced on Illumina PE 250.

### 2.6. Bioinformatics Analysis

The microbial data were qualified by Usearch (version 7.1 http://drive5.com/uparse/). The phylogenetic affiliation of all 16S rRNA genes sequence were adjusted with the SILVA reference database (Release119 http://www.arb-silva.de), and allocated into operational taxonomic units (OTUs) (97% similarity threshold). Finally, data were categorized with the RDP classifier (version 2.2 http://sourceforge.net/projects/rdp-classifier/). Chimeric sequences was removed with USEARCH software (version 7.1 http://drive5.com/usearch/manual/singletons.html). In the current research, venn was used to compute the OTUs in each treatment. Beta diversity indexes, such as nonmetric multidimensional scaling (NMDS), were analyzed to evaluate the diversity of species complexity. Enriched OTUs of gut microbe were first compared the relative abundance of OTUs with the non-parametric factorial Kruskal–Wallis (KW) sum-rank test (*p <* 0.05).

### 2.7. Statistical Analysis

The data for non-omics were statistically analyzed using one-way ANOVA by Tukey test with SPSS 20.0 (SPSS Inc., Chicago, IL, USA). The normal distribution was detected by the Kolmogorov-Smirnov procedure. Normalized data were analyzed with one-way ANOVA following Tukey test [22], and Non-normally distributed data were analyzed by the Kruskal-Wallis test with Duncan’s multiple comparison tests [22] for the date of omics. Plotting was placed with GraphPad Prism 7.0 (GraphPad Software Inc., San Diego, CA, USA). Values were expressed as means ± standard deviation (SD). The significant differences were considered at a probability level of *p <* 0.05.

## 3. Results

### 3.1. F Residues in the Duodenum of Laying Hens

**Figure 1:**
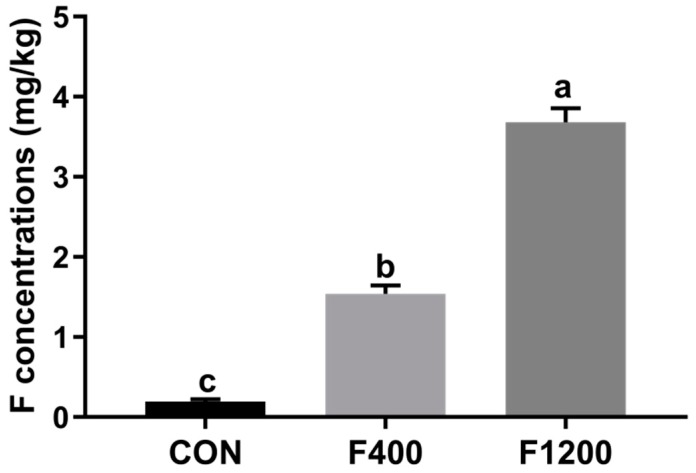
Effects of dietary fluoride (F) level on the contents of F in the duodenum. Values were showed as the means and SD (*n* = 6). a–c Means with different superscript letters are significantly different (*p* < 0.05). CON, the control group, basal diet; F400 and F1200, diets supplemented with 400, and 1200 mg F/kg feed from sodium fluoride, respectively.

Figure 1 showed that the F residues in the duodenum of laying hens were increased significantly with the increase of dietary F supplemental levels (*p* < 0.05). Dietary F levels at 400 to 1200 mg/kg accelerated the F deposition in the duodenum significantly (*p* < 0.05).

### 3.2. Digestive Enzyme Activities

**Figure 2:**
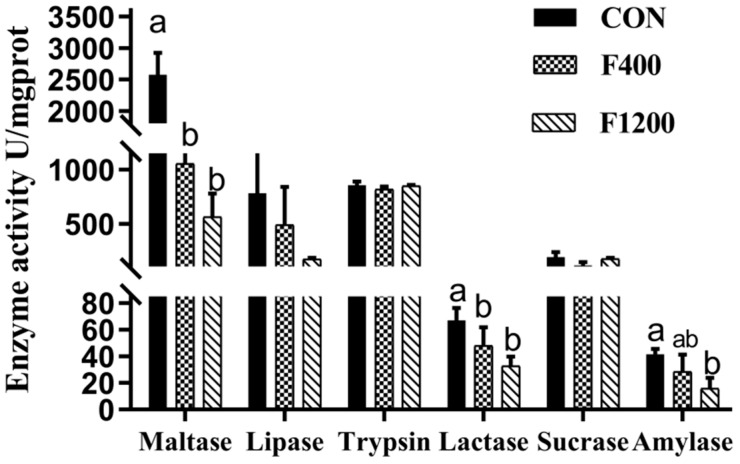
Effects of dietary fluoride levels on digestive enzyme activities of laying hens (*n* = 6). Values were showed as the means and SD (*n* = 6). ^a,b^ Means without common letters above the histogram differ significantly (*p* < 0.05). CON, the control group, basal diet; F400 and F1200, diets supplemented with 400, and 1200 mg F/kg feed from sodium fluoride, respectively.

Date from Figure 2 showed that high dietary F levels decreased (*p* < 0.05) the activities of amylase, maltase and lactase. However, there is no significant difference in the activities of lipase, trypsin and sucrase (*p* > 0.05). The data in amylase activity showed that dietary F level at 1200 mg/kg was more powerful potent than at 400 mg/kg.

### 3.3. MUC2 and sIgA mRNA Expression

**Figure 3:**
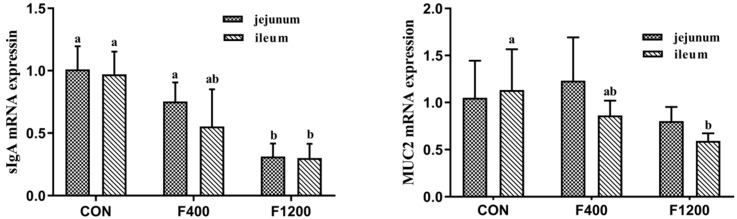
Effect of dietary fluoride levels on the mRNA expression levels of sIgA and MUC2 in jejunum and ileum of laying hens (*n* = 6 in each group). Values were showed as the means and SD (*n* = 6). (**a**,**b**) Means without common letters above the histogram differ significantly (*p* < 0.05). sIgA, Secretory immunoglobulin A; MUC2, Mucin 2. CON, the control group, basal diet; F400 and F1200, diets supplemented with 400, and 1200 mg F/kg feed from sodium fluoride, respectively.

The abundance of MUC2 and sIgA mRNA in the jejunum and ileum of laying hens after feeding 49d was shown in Figure 3. Intestinal sIgA mRNA expression levels were lower (*p* < 0.05) in hens fed 1200 mg/kg F diet, compared with the control group. MUC2 mRNA expression levels were significantly decreased (*p* < 0.05) only in the ileum of hens fed 1200 mg/kg F diet, and did not differ in the jejunum.

### 3.4. Concentrations of sIgA in Jejunum and Ileum

**Figure 4:**
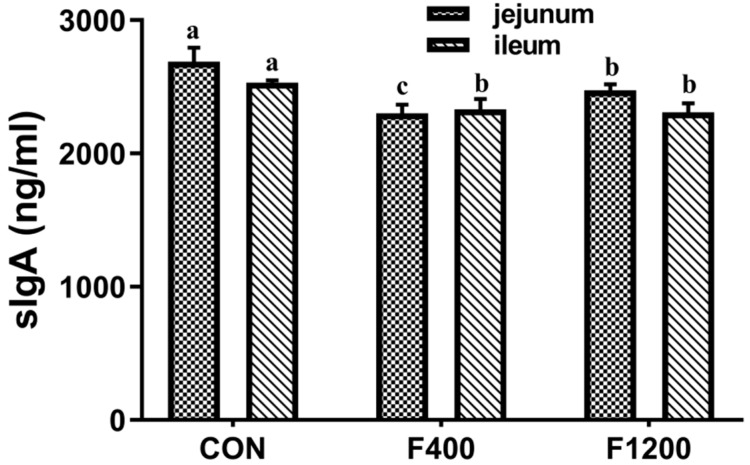
Effect of dietary fluoride levels on the contents of sIgA in jejunum and ileum of laying hens (*n* = 6 in each group). Values were showed as the means and SD (*n* = 6). (**a**–**c**) Mean without common letters above the histogram differ significantly (*p* < 0.05). sIgA, Secretory immunoglobulin A. CON, the control group, basal diet; F400 and F1200, diets supplemented with 400, and 1200 mg F/kg feed from sodium fluoride, respectively.

The concentrations of sIgA in the jejunum and ileum of laying hens were shown in Figure 4. There were significant decreases (*p* < 0.05) in intestinal sIgA levels in laying hens fed 1200 mg/kg F diet, compared with the control diet in jejunum and ileum; besides a decrease between the control group and F400 group was also detected in jejunum; however, no significant differences were detected between the control and the F400 groups in ileum (*p* > 0.05).

### 3.5. Cecal Microbiota

**Figure 5:**
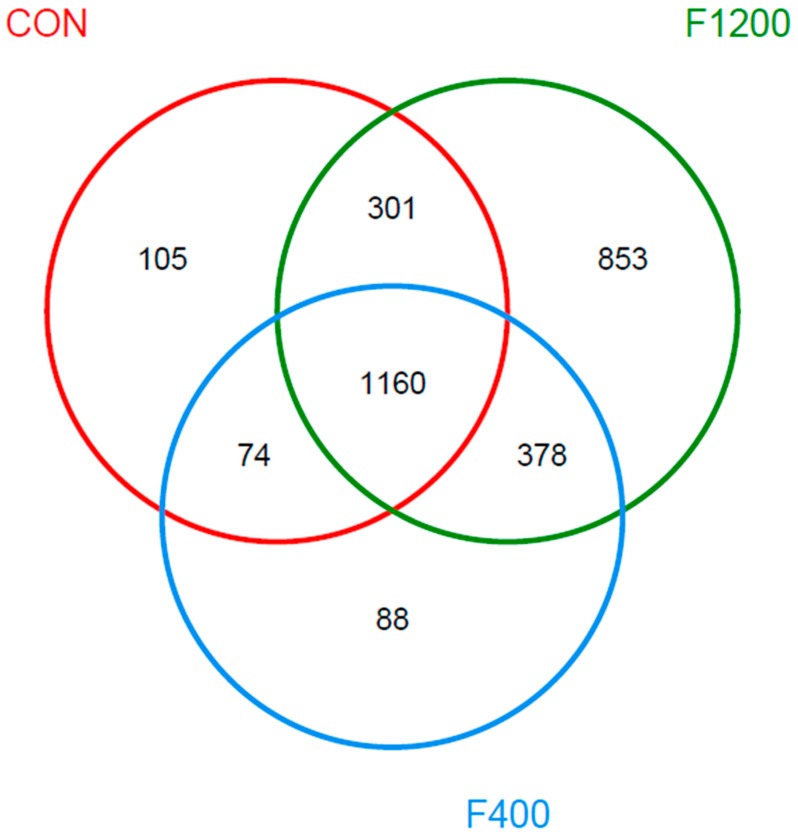
Effects of dietary fluoride level on Venn diagram of cecal microbiota between groups (*n* = 5). Venn diagram was obtained at operational taxonomic units (OTUs) levels (97% similarity threshold). CON, the control group basal diet; F400 and F1200, diets supplemented with 400, and 1200 mg F/kg feed from sodium fluoride, respectively.

The observed OTUs of three treatments were presented as Venn diagram in Figure 5. Total of 2959 OTUs were detected in the present study, 105 uniquely vested in the control group, 88 in the low-F group, while 853 in the high-F group.

**Figure 6:**
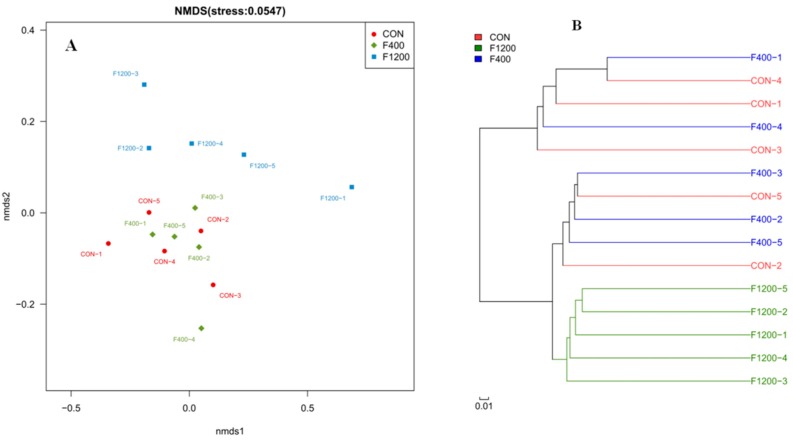
Effects of dietary fluoride level on the beta diversity of cecal microbiota between groups (*n* = 5). (**A**) nonmetric multidimensional scaling (NMDS) plot. (**B**) Clustering analysis. CON, the control group, basal diet; F400 and F1200, diets supplemented with 400, and 1200 mg F/kg feed from sodium fluoride, respectively

As shown in Figure 6, the samples of the control and F400 groups were clustered with each other, while well separated from the F1200 group by NMDS and clustering analysis. These results suggested that the composition of cecal microbiota in laying hens was changed by high dietary F level.

**Figure 7:**
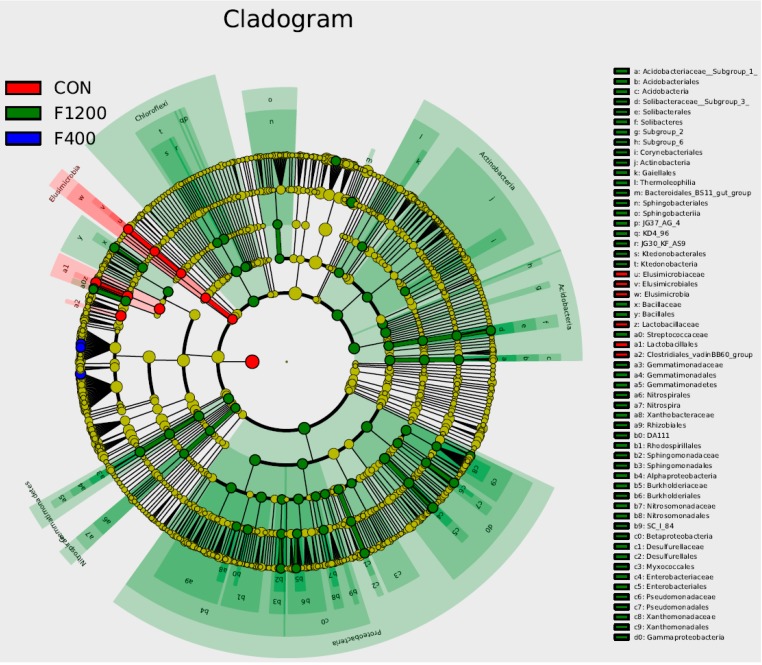
Cladogram representation of the differentially abundant families and genera (only top 50% are plotted here, *n* = 5). The root of the cladogram indicates the domain bacteria. The taxonomic levels of phylum and class are marked, while family and genus are abbreviated, with different colors indicating the group hosting the greatest abundance. The size of each node represents its relative abundance. CON, the control group, basal diet; F400 and F1200, diets supplemented with 400, and 1200 mg F/kg feed from sodium fluoride, respectively.

LEfSe analysis of family and genus level abundance was shown in Figure 7. It could be observed that *Lactobacillus* was biomarkers in the control group, while *Gammaproteobacteria*, *Streptococcaceae*, *Enterobacteriales,* and *Enterobacteriaceae*, which are known to include (potential) pathogens of poultry and humans, were biomarkers of the F1200 group.

**Figure 8:**
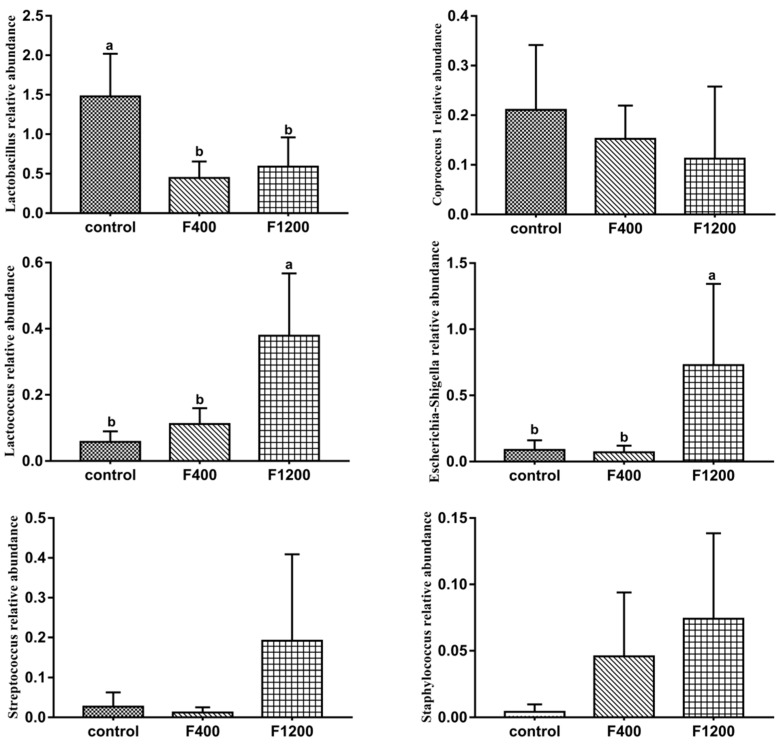
Effects of dietary fluoride level on the relative abundance of intestinal opportunistic pathogen and beneficial bacteria in cecum of laying hens. Values are means ± SD (*n* = 5). a–b Means with different superscript letters are significantly different (*p* < 0.05). CON, the control group, basal diet; F400 and F1200, diets supplemented with 400, and 1200 mg F/kg feed from sodium fluoride, respectively.

The change of the relative abundance of some pathogenic bacteria and beneficial bacteria was presented in Figure 8. The abundance of *Lactococcus* and *Escherichia-Shigella* were expanded (*p < 0.05*) significantly, while *Lactobacillus* was lower (*p <* 0.05) and *Escherichia-Shigella* was higher (*p <* 0.05) in the F1200 treatment than those of the other two groups.

## 4. Discussion

The present study was taken out to evaluate the effects of dietary F levels on digestive enzymes activities, intestinal mucosal immunity, and ceca microbial community of laying hens. When taking out the feeding trial, we observed that the daily feed intake of laying hens in the dietary high F group was gradually decreasing with the test time, accompanied by watery stools. Subsequently, the egg production of hens fed with high F diets was significantly decreased [23]. In the present study, we detected that with the increased dietary F, the contents of F in the duodenum were significantly increased. Based on the morphological examination, it was found that the small intestine structure and mitochondria of enterocyte were destroyed in the dietary high F groups (supplementary material Figures S1 and S2, and Table S1, Miao, et al. 2019, unpublished). Mitochondria, double-membrane-bound subcellular organelles, play a vital role in metabolic functions with providing energy production [24]. In early events of thymocyte apoptosis, it was observed that the mitochondrial structure was altered and swollen [25]. Previous research reported that chronic fluoride exposure resulted in mitochondrial structural alterations (mitochondrial cristae disorder and mitochondrial membrane potential loss) in rat brain and cultured neurons [26,27]. Correspondingly, in the current research, we found that dietary high F level lowered the activities of amylase, maltase and lactase in the duodenum of laying hens. However, there is no significant difference in the activities of lipase, trypsin and sucrase. These results indicated that dietary high F level mainly reduced the starch digestion and absorption rather than the protein and fat. This is agreement with the previous research found that hens with low-energy intake produced fewer eggs than hens fed the high-energy diet [28]. We speculated that the lower laying performance of hens fed with dietary high F levels was related to the decreased activities of digestive enzymes, and high F contents in the duodenum resulted in the destruction of small intestine structure and mitochondria of laying hens, which may contribute together to the decrease of digestive enzyme activities in the duodenum of hens fed high F diets. As far as we know, there is no research about the effects of dietary F levels on digestive enzymes activities of hens.

The mucus layer overlying the small intestinal epithelium secreted by goblet cells and Paneth cells [29], promotes the elimination of gut contents and is known as the first line of defense against physical and chemical injury caused by ingested food, microbe and the microbial products [30,31]. The major protein components of small intestinal mucus layer are MUC2, sIgA, IgG, IgM, and nonspecific antimicrobials [31]. MUC2 mucin secreted by goblet cells is the major component of the intestinal mucus barrier in the apical membrane [32]. Colonic inflammation was determined in the mouse with missense mutations Muc2 gene [33]. In the current study, expression of ileum MUC2 mRNA was decreased by dietary high F concentration in laying hens. In chickens, as in mammals, there exists a separate mucosal immune system in the gut responding differently in many ways from the systemic immune system [34], one of which is the sIgA production in the intestinal immune responses [35]. In the small intestine, the sIgA antibody is synthesised by plasma cells in the lamina propria, and then selectively translocated through intestinal epithelial cells and transferred to the intestinal lumen [34,36]. In addition, sIgA modulates the composition of the microbiota and contributes to the maintenance of homeostasis, so that the absence of sIgA may lead to inflammation [37]. In the current study, expression of jejunum and ileum sIgA mRNA was decreased by high dietary F concentration in laying hens. Correspondingly, decreases in jejunum and ileum sIgA concentrations of hens fed a high F diet were detected in this study. It was suggested that dietary high F may simultaneously have transcriptional and post-transcriptional regulation for sIgA genes in laying hens; however, the detailed mechanism needs to further investigate. It was speculated that the decreases of sIgA and MCU2 is relative to the damage of the structure of intestinal epithelium and mitochondrial dysfunction. Moreover, the decrease promoted the development of intestinal inflammation, increased the chance of pathogen invasion, and reduced disease resistance.

The present study compared the difference of cecal bacterial communities of hens with different dietary F intake. According to our determined samples, dietary high F level increased the bacterial richness and diversity of laying hens, while dietary low F level would not change it (Miao, et al. 2019, unpublished). In the current study, the microbial communities of hens fed basal and dietary low F diets were similar, while different from those fed high F diet. The results suggested that the composition of cecal microbiota in laying hens was changed by dietary high F intake. *Lactobacillus* was higher in the control group, while *Gammaproteobacteria*, *Escherichia-Shigella*, *Enterobacteriaceae*, *Streptococcus,* and *Staphylococcus*, which are known to include (potential) pathogens of poultry and humans, were higher in high-F group. These results were agreement with the report of Luo et al. [12] on the effects of F on the broiler chicken ileum and cecal microbiota, which found that compared with those in the control group, the counts of *Lactobacillus* spp. was decreased, and the counts of *Escherichia coli* (*E-coli*) and *Enterococcus* spp. were significantly increased in the high F groups (800 mg/kg and 1200 mg/kg F). *Lactobacillus* is a beneficial commensal for humans and animals [38], and believed that the enriched *Lactobacillus* could protect the gut from pathogens and promote efficient nutrient and energy extraction [39]. In the current study the relative abundances of *Streptococcus* and *Staphylococcus* were higher in the high-F groups than those in the control treatment. Staphylococci produce multiple toxins and enzymes, such as cytotoxins, protease, and lipase, which result in proinflammatory changes in cells and destroy tissues [40]. The genus *Streptococcus* includes extensive Gram-positive and coccus-shaped organisms, which include many opportunistic pathogens [41]. *Enterobacteriaceae* is a large family of bacteria that is closely related to the intestines of human and birds and enteric pathogens, such as *Escherichia coli*, *Shigella*, *Salmonella* belong to this family [42]. *Shigella* is a gram-negative enteroinvasive bacterium responsible for bacillary dysentery in constrained hosts [43]. Previous study found that *Shigella* infected the chicken intestines through invading the intestinal mucosa and caused pathogenicity, and even death [44]. In the current study, watery stools and mortality were heavier in high-F group, which may be owned to the high relative abundance of *Escherichia-Shigella* in the ceca. *Gammaproteobacteria* includes (potential) pathogens of poultry and humans. Such as, *E-coli* are a *Gammaproteobacteria* and some *E-coli* strain can result in opportunistic infections in poultry [45]. These results indicated that the hens fed high-F diets were more susceptible to attack by pathogenic microorganisms than those fed basal and low-F diets.

## 5. Conclusions

In summary, the current study revealed that the concentrations of F in duodenum were increased with the increase of dietary F levels, and dietary high F level decreased the digestive enzymes activities, reduced the mucosal immunity, and caused the enteric dysbacteriosis of laying hens.

## Figures and Tables

**Table 1:** Ingredient and nutrient composition of the basal diet (air-dry basis).

Ingredients	Composition, %	Nutrient ^2^	Composition, %
Corn	65.00	Metabolizable energy, Mcal/kg	2.65
Soybean meal (44.20% crude protein)	20.50	Crude protein, %	15.64
Fish meal	2.50	Calcium, %	3.51
Limestone	7	Total phosphorus, %	0.65
		Available phosphorus, % Available phosphoru	0.46
Premix ^1^	5.00	Lysine, %	0.82
		Methionine, %	0.36
		Tryptophan, %	0.17
Total	100.00	(Methionine + cysteine), %	0.65

^1^ The premix provided the following per kg of the diet: vitamin A, 7600 IU; vitamin D3, 2000 IU; vitamin E, 15 IU; vitamin K, 2 mg; thiamine, 1 mg; riboflavin, 8.5 mg; calcium pantothenate, 50 mg; niacin, 32.5 mg, pyridoxine, 8 mg; biotin, 2 mg; folic acid, 5 mg; vitamin B12, 5 mg; choline, 500 mg; Mn, 65 mg; I, 1 mg; Fe, 65 mg; Cu, 10 mg; Zn, 66 mg; Se, 0.12 mg. ^2^ Estimated from Chinese feed database provided with tables of feed composition and nutritive values in China (2015 twenty-sixth edition).

**Table 2:** Primer sequences used for RT-qPCR analysis (F: forward; R: reverse).

Gene Symbol	Gene Name	Primer Sequence (5′-3′)	Accession No.
18s rRNA^1^	18s rRNA	F: ATTCCGATAACGAACGAGACT R: GGACATCTAAGGGCATCACA	AF173612.1
MUC2	Mucin 2	F: CAGCACCAACTTCTCAGTTCC R: TCTGCAGCCACACATTCTTT	NM_001318434
sIgA	Secretory immunoglobulin A	F: ACCACGGCTCTGACTGTACC R: CGATGGTCTCCTTCACATCA	S40610

18s rRNA worked as endogenous reference gene.

## Supplementary Materials

The following are available online at https://www.mdpi.com/2076-2615/10/2/179/s1, Figure S1: Effect of dietary fluoride (F) concentrations on small intestinal morphology of laying hens (*n* = 6), Figure S2: Transmission electron microscopy micrographs from small intestine of laying hens fed basal or fluoride diet, Table S1: Mean values (±standard deviation, SD) of small intestinal morphology of laying hens exposed to fluoride.
