# Supplementary material for: Dietary High Sodium Fluoride Impairs Digestion and Absorption Ability, Mucosal Immunity, and Alters Cecum Microbial Community of Laying Hens
Source: Animals (Basel). 2020 Jan 21;10(2):179. doi: 10.3390/ani10020179 (PMC7070338; doi:10.3390/ani10020179)
Supplement: Supplementary file 1 [file animals-10-00179-s001.pdf]

Supplementary Materials

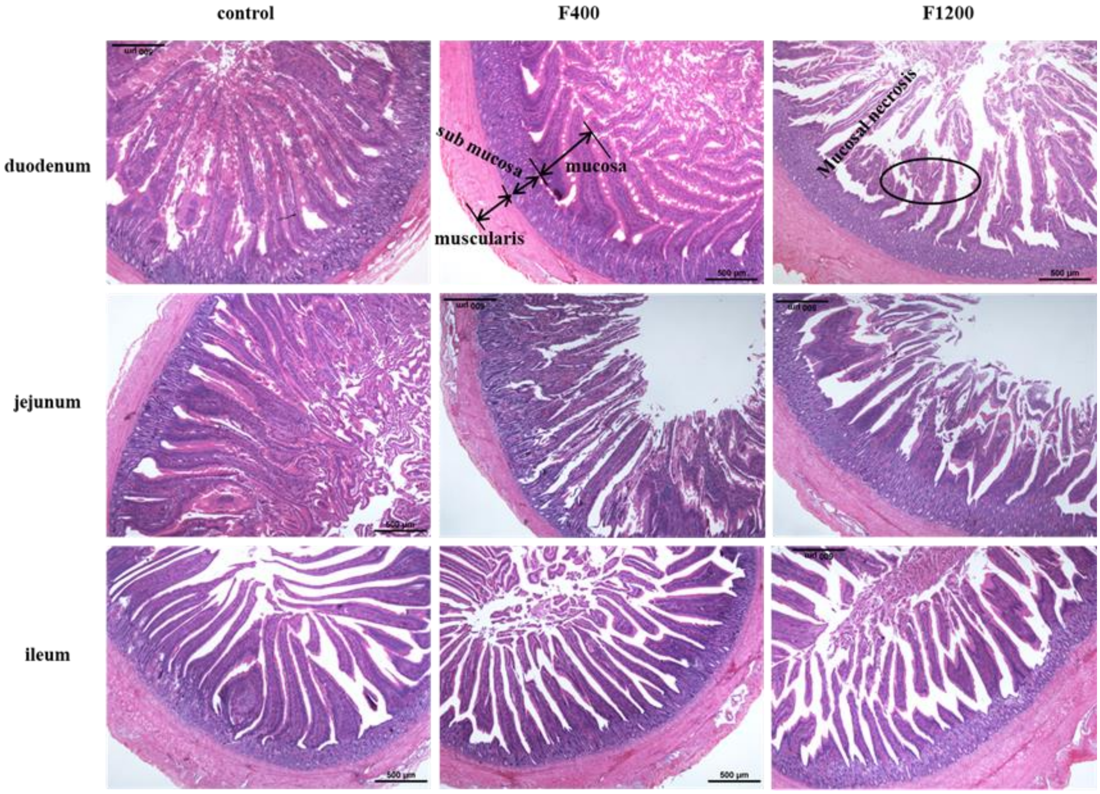

Figure S1. Effect of dietary fluoride (F) concentrations on small intestinal morphology of laying hens (n = 6). Hematoxylin and eosin (H and E) staining (4×), scale bar 500 μm. Control, basal diet; F400 and F1200, diets supplemented with 400, and 1200 mg F/kg feed from sodium fluoride, respectively.

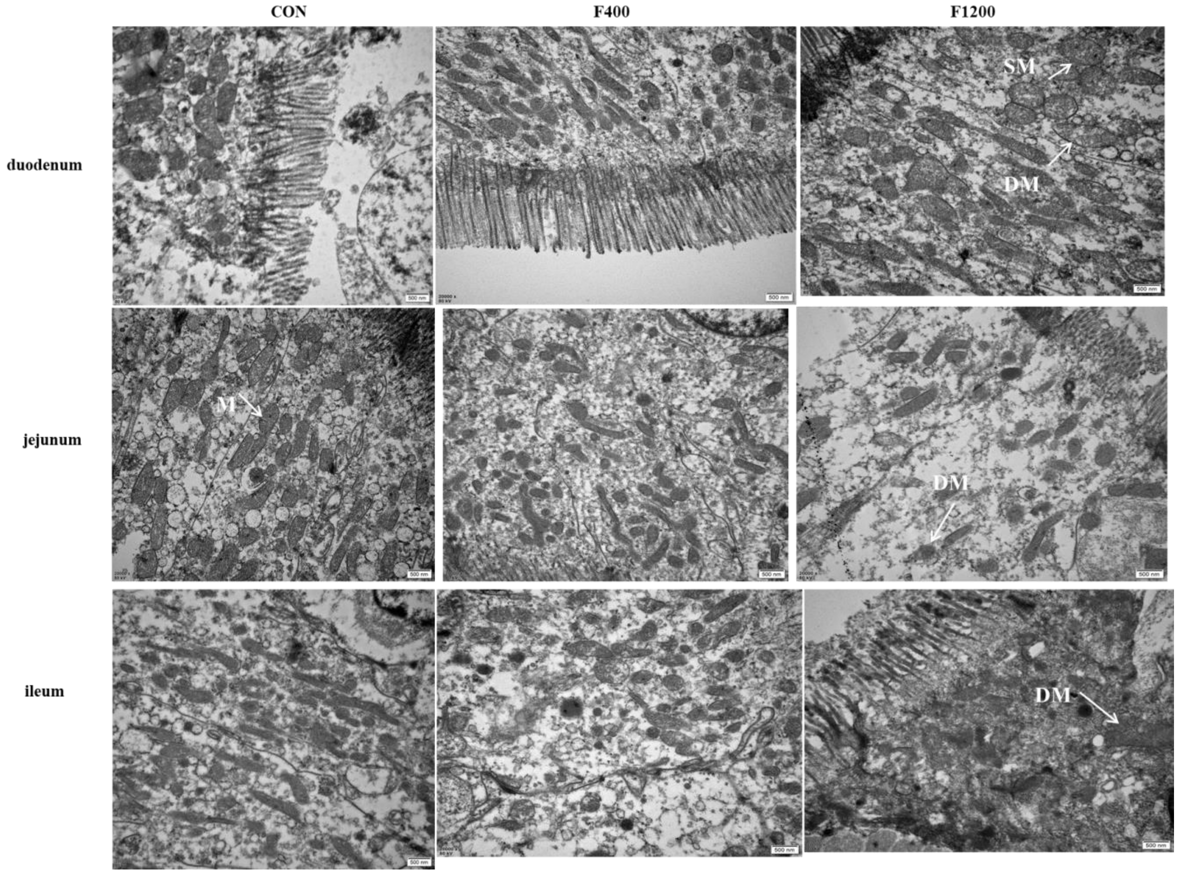

Figure S2. Transmission electron microscopy micrographs from small intestine of laying hens fed basal or fluoride diet. M: mitochondrion, SM: swollen mitochondria, DM: dissolved membrane with cristae disorders. Bar 500 µm. CON, basal diet; F400 and F1200, diets supplemented with 400, and 1200 mg F/kg feed from sodium fluoride, respectively.

Table S1. Mean values (±standard deviation, SD) of small intestinal morphology of laying hens exposed to fluoride.

Item		Treatment		
		CON	F400	F1200
Duodenum	Villus height(mm)	1.52 ± 0.09[a]	1.18 ± 0.17[b]	0.93 ± 0.13[c]
	Villus area(mm^2)	0.20 ± 0.01[a]	0.19 ± 0.01[a]	0.10 ± 0.01[b]
	V/C	5.41 ± 0.38[a]	4.74 ± 0.46[a]	2.50 ± 0.40[b]
	Crypt depth(μm)	264.65 ± 26.94[b]	281.82 ± 47.01[b]	344.35 ± 33.63[a]
	Villus width(μm)	269.11 ± 24.01[a]	222.87 ± 22.83[b]	144.26 ± 9.43[c]
Jejunum	Villus height(mm)	1.42 ± 0.07[a]	1.18 ± 0.06[b]	0.99 ± 0.06[c]
	Villus area(mm^2)	0.21 ± 0.03[a]	0.15 ± 0.02[b]	0.12 ± 0.01[c]
	V/C	6.04 ± 1.12[a]	5.59 ± 1.54[ab]	4.35 ± 0.55[b]
	Crypt depth(μm)	219.28 ± 18.75[b]	201.37 ± 16.60[b]	263.28 ± 11.90[a]
	Villus width(μm)	213.33 ± 22.87[a]	182.21 ± 18.90[b]	131.51 ± 14.66[c]
Ileum	Villus height(mm)	1.20 ± 0.07[a]	1.01 ± 0.08[b]	1.02 ± 0.12[b]
	Villus area(mm^2)	0.15 ± 0.02[a]	0.13 ± 0.03[ab]	0.11 ± 0.03[b]
	V/C	5.94 ± 1.19	5.70 ± 0.86	5.79 ± 1.20
	Crypt depth(μm)	206.48 ± 44.42	207.88 ± 24.78	188.00 ± 10.05
	Villus width(μm)	152.88 ± 27.49	149.66 ± 17.59	141.78 ± 27.17

V/C, the ratio of villus height to crypt depth. Values without common letters in a row differ significantly (one-way ANOVA, α = 0.05, n = 6). CON, basal diet; F400 and F1200, diets supplemented with 400, and 1200 mg F/kg feed from sodium fluoride, respectively.
